# Spatio-temporal patterns of lumpy skin disease outbreaks in dairy farms in northeastern Thailand

**DOI:** 10.3389/fvets.2022.957306

**Published:** 2022-08-04

**Authors:** Veerasak Punyapornwithaya, Suvaluk Seesupa, Sitthinon Phuykhamsingha, Orapun Arjkumpa, Chalutwan Sansamur, Chaiwat Jarassaeng

**Affiliations:** ^1^Center of Excellence in Veterinary Public Health, Faculty of Veterinary Medicine, Chiang Mai University, Chiang Mai, Thailand; ^2^Veterinary Public Health and Food Safety Centre for Asia Pacific (VPHCAP), Faculty of Veterinary Medicine, Chiang Mai University, Chiang Mai, Thailand; ^3^Faculty of Veterinary Medicine, Khon Kaen University, Khon Kaen, Thailand; ^4^Khon Kaen Dairy Cooperative, Khon Kaen, Thailand; ^5^Department of Livestock Development, Animal Health Section, The 4th Regional Livestock Office, Khon Kaen, Thailand; ^6^Akkhraratchakumari Veterinary College, Walailak University, Nakhon Si Thammarat, Thailand

**Keywords:** lumpy skin disease, outbreak, cluster, spatio-temporal pattern, dairy cattle, Thailand

## Abstract

In 2021–2022, there were numerous outbreaks of lumpy skin disease (LSD) affecting cattle farms across Thailand. This circumstance was the country's first encounter with an LSD outbreak. Thus, a better understanding of LSD epidemiology is necessary. The aim of this study was to determine the spatio-temporal patterns of the LSD outbreaks in dairy farming areas. Data from LSD outbreak investigations collected from dairy farms in Khon Kean province, northeastern Thailand, were analyzed using spatio-temporal models including space-time permutation, Poisson, and Bernoulli models. LSD outbreaks were found in 133 out of 152 dairy farms from May to July, 2021. The majority of dairy farms (*n* = 102) were affected by the LSD outbreaks in June. The overall herd attack, morbidity and mortality rates were 87, 31, and 0.9%, respectively. According to the results of all models, the most likely clusters were found in the northern part of the study area. The space-time permutation and Poisson model identified 15 and 6 spatio-temporal outbreak clusters, respectively, while the Bernoulli model detected only one cluster. The most likely clusters from those models cover radii of 1.59, 4.51, and 4.44 km, respectively. All farms included in the cluster identified by the space-time permutation model were also included in the cluster identified by the Poisson model, implying that both models detected the same outbreak area. Furthermore, the study results suggested that farmers who own farms within a one km radius of the LSD outbreak farm should be advised to implement more stringent insect vector control measures to prevent disease spread. This study provides better insights into the spatio-temporal pattern of clusters of LSD in the outbreak area. The findings of this study can support authorities in formulating strategies to prevent and control future outbreaks as well as prioritizing resource allocation to high-risk areas.

## Introduction

According to the World Organization for Animal Health (WOAH), lumpy skin disease (LSD) is an emerging transboundary notifiable viral disease (1). This disease is caused by LSD virus (LSDV), a *capripoxvirus* within the *Poxviridae* family (2). The primary route of LSD transmission is driven by insect vectors such as stable files (3, 4) and mosquitoes (5, 6). Originating from the African continent, LSD has been reported in several countries in Europe, the Middle East and Asia (7). Currently, several Asian countries, including Bangladesh (8), China (9), India (10), Bhutan, Nepal (11), Vietnam (12), Myanmar (13), Hong Kong (14), Taiwan (15), Thailand (16) and Malaysia (17), are affected by this disease. The economic consequences of LSD are substantial, as the disease has a remarkable negative financial impact on cattle herd owners, customers, and the livestock industry (18, 19).

In Thailand, the first outbreak of LSD was found in beef farms located in the northeastern part of the country in March 2021 (16). Later, numerous LSD outbreaks were reported across the country (20, 21). Data collected by livestock authorities as part of the LSD outbreak investigation and control program in March 2022 revealed that the total number of beef and dairy cattle affected by LSD was 609,073, and 12,317, respectively (22). During the outbreaks, both beef and dairy cattle were affected by LSD, which resulted in poor health and decreased production (23).

Scan statistical methods (24) are increasingly being used in epidemiological research with the aim of detecting clusters in the spatial, temporal, or spatio-temporal aspects of infectious disease outbreaks (25–27). In purely spatial or temporal analysis, a scanning window moves across space or time to detect disease clusters within the target area whereas a cylinder moves both spatially and temporally to detect potential spatio-temporal clusters (28, 29). Space-time permutation, Poisson, and Bernoulli models are the most commonly used scan statical methods. These methods require different data inputs and have their own computation procedures. All models necessitate the use of geographic coordinate data. The number of cases is used in the space-time permutation model (STP), whereas the Poisson model requires both the number of cases and the total number of populations at risk (30). The Bernoulli model uses data from both cases and controls as its input data (24). Normally, these models are employed to determine clusters with a high rate of infection. The identified clusters can be thought of as a “hot zone” for disease outbreaks. Thus, intervention and resource allocations in these areas can be prioritized (24, 30).

Given that LSD was only recently discovered in the country, a better understanding of the epidemiology of LSD is necessary. However, there are very few reports on the epidemiology of LSD in Thailand. Even though a study of the spatio-temporal pattern of the outbreak in beef cattle farms was carried out (23), studies on the epidemiology of LSD outbreaks in dairy farms remain limited, allowing several knowledge gaps, particularly on how the LSD outbreaks distributed among dairy farms in terms of space and time. The findings of the study in beef farming areas (23) may not be extrapolated to dairy farming areas because beef and dairy cattle farms in Thailand are managed differently (31, 32). Furthermore, the spatial distributions of beef and dairy farms differ, with the majority of dairy farming areas being densely populated with dairy farms and beef farms being sparsely populated. Thus, it is essential to investigate spatio-temporal patterns of LSD outbreaks in dairy farming areas in order to gain a better understanding of the spatial epidemiology of LSD outbreaks affecting dairy farms.

The objective of this study was to determine spatio-temporal patterns of LSD outbreaks in dairy farms in northeastern Thailand using the STP, Poisson and Bernoulli models.

## Materials and methods

### Study area and lumpy skin outbreak data

Outbreaks of LSD were reported in dairy farms located in dairy farming areas in Muang and Ban Haet districts, Khon Kean Province between May to July 2021. There was no prior history of LSD outbreaks in any dairy farms in these areas. The dairy farms are operated by dairy farmers who are members of the Khon Kean dairy cooperative. All dairy farms use a free-stall system; therefore, there is no chance of dairy cattle from one farm interacting with those from other farms.

During the outbreak, licensed veterinarians investigated the outbreak at every dairy farm in the outbreak areas with the support of the dairy cooperative committee. Additionally, the investigations were supervised by livestock authorities from the Khon Kean provincial livestock office, Department of Livestock Development (DLD).

All dairy cattle on each farm that participated in the study were examined by veterinarians for clinical signs of LSD. Similar to a previous report (23), an LSD animal case was defined as a dairy cow displaying the LSD clinical signs, which include raised firm nodules with a diameter of 1–7 cm on the head and/or body surface areas. Notably, veterinarians conducting outbreak investigations did not collect animal samples to confirm LSD infections. However, in order to verify this LSD outbreak, livestock authorities from the DLD collected blood and tissue samples from some dairy cattle from various farms in the outbreak areas. Thereafter, samples were processed in a laboratory by the National Institute of Animal Health (NIAH), which is part of the DLD. After that, the laboratory-confirmed results were made available to the public on the DLD website (22).

During the investigation, basic epidemiological data were collected, including the number of dairy cattle showing LSD clinical signs, the number of dairy cattle showing LSD clinical signs and then dying, and the number of all dairy cattle on the farm. The authorities also recorded the geographical coordinates of each farm in the study area.

In the present study, we analyzed data from such outbreak investigations. For each LSD outbreak farm, morbidity and mortality rates at the animal level were calculated. The herd attack rate was also determined to investigate how LSD affected all dairy farms in the study area. Several maps were created to present geographical information relevant to the LSD outbreaks. All maps were created by Quantum Geographic Information System (QGIS), which is an open-source software (33).

### Spatio-temporal analysis

In addition to geographical coordinate data, the availability of (i) number of cases, (ii) number of cases and population at risk and (iii) number of cases for LSD outbreak farms and non-LSD outbreak farms (control farm) allows data to be analyzed in the dimensions of space and time using SPT, Poisson and Bernoulli models (34), respectively.

### Space-time permutation model

The SPT model evaluates LSD case data within each cylindrical window and calculates the ratio of the observed number of LSD cases to the expected number of LSD cases. The likelihood ratio test statistic was then determined from each scanning circular window. A cluster is detected when an elevated number of LSD cases are found within an area and within a certain time frame compared to the rest of the study area for the same time frame (28).

### Poisson model

The Poisson model evaluates the number of dairy cattle populations at risk and the number of LSD cases within and outside the cylindrical windows in the dimensions of space and time. For each cylinder, the relative risk and log likelihood ratio (LLR) were calculated. The most likely cluster was determined to be the window with the maximum LLR value and a *p*-value <0.05.

For a given scanning window, the LLR is determined based on the following equation (35):


$$\begin{array}{l} {\left(\frac{n}{E}\right)}^{n}{\left(\frac{N-n}{N-E}\right)}^{N-n}I\left(n>E\right) \end{array}$$

where n is the number of LSD cases within the scan window, *N* is the total number of dairy cattle on each farm, *E* is the expected number of LSD cases under the null hypothesis, and *I* denotes the indicator function (36).

### Bernoulli model

The Bernoulli model used information from both LSD outbreak farms and control farms to detect spatio-temporal clusters. Similar to the Poisson, the LLR was calculated for each cylindrical window, and the cluster with the maximum LLR with a *p*-value <0.05 was justified as the most likely cluster. The likelihood function of this model is written as:


$$\begin{array}{l} {\left(\frac{c}{n}\right)}^{c}{\left(\frac{n-c}{n}\right)}^{n-c}{\left(\frac{C-c}{N-n}\right)}^{C-c}\\ {\left(\frac{\left(N-n\right)-\left(C-c\right)}{N-n}\right)}^{\left(N-n\right)-\left(C-c\right)}I\left(\right) \end{array}$$

where *C* is the total number of LSD cases, *c* is the observed number of LSD cases within the window, *n* denotes the total number of LSD cases, *N* represents the combined total number of cases and controls within the dataset, and *I*() denotes the indicator of function *I*, which is equal to 1 in the case of *c*>*C*/*N* or () otherwise.

In the present study, SaTScan software was used to identify LSD spatio-temporal clusters (30). For parameter setting across all models, the maximum spatial cluster size was set as 50% of the population at risk, which is the default setting value. With this setting, a clusters size can contain at most 50% of the total population at risk (30). The maximum temporal cluster size was set at 7 days based on the LSD incubation period (7) and those used in the previous study (23). To determine the significance of candidate clusters, Monte Carlo simulation was employed (number of replications = 999). The clusters with *p*-value < 0.05 were considered as statistically significant clusters. Moreover, maps corresponding to the SaTScan results were created using QGIS software.

## Results

### Epidemiological and spatial features

**Figure 1:**
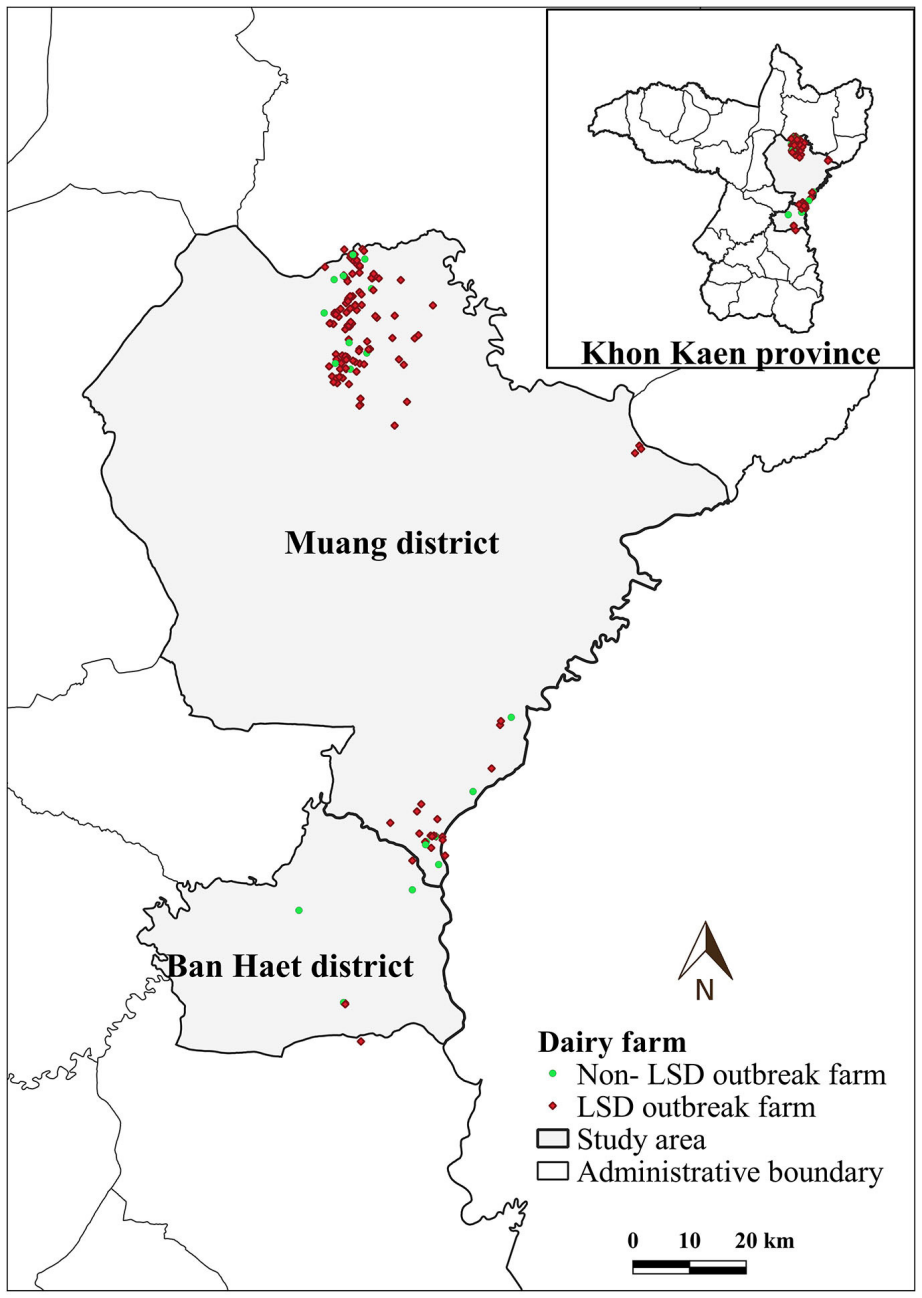
Study area, farm location and farm status based on lumpy skin disease (LSD) affecting status.

**Figure 2:**
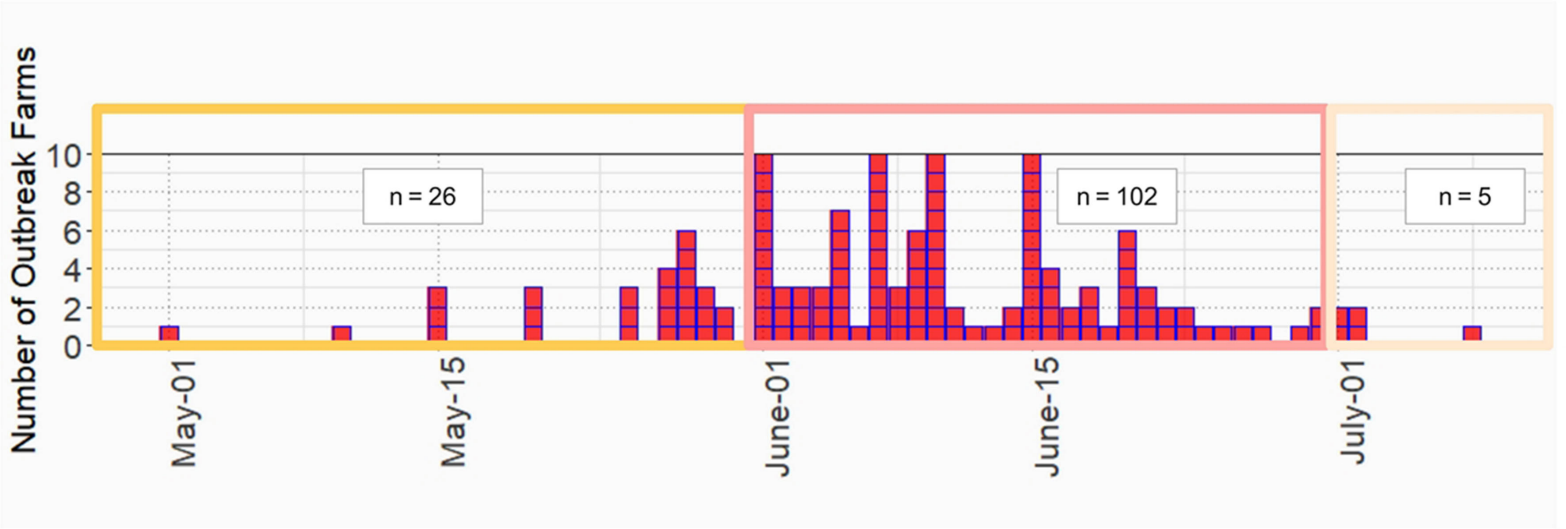
Epidemic curve of lumpy skin disease outbreak in dairy farms, Khon Kean province, Thailand.

LSD outbreaks were found in 133 out of 152 dairy farms from May 1st to July 8th in the year 2021. The locations of dairy farms with and without LSD outbreaks are depicted in Figure 1. The herd attack rate was 87%. Overall, the morbidity and mortality rates were 31% (*n* =2,656/8,553) and 0.9% (*n* = 77/8,553), respectively. According to the epidemic curve, the highest number of LSD outbreaks were found in June followed by July. In June, more than 100 dairy herds were affected by LSD. Additionally, on several days in June, as many as ten farms reported LSD outbreaks (Figure 2).

The average total number of dairy cattle in dairy farms enrolled in this study was 57 heads. The average distance between a dairy farm and its nearest neighbor farm was 0.83 km (range: 0.12–7.31 km). Notably, in some areas, numerous farms are aggregated (Figure 1).

### Spatio-temporal clusters

The most likely clusters detected by all models were located in the northern part of Muang district during the period of June, 2021. The STP model identified 16 clusters including the most likely cluster and 15 secondary clusters (Table 1) while the Poisson model yielded the most likely cluster and 6 secondary clusters (Table 2). The Bernoulli model detected only the most likely clusters (Table 3). Furthermore, the most likely cluster defined by the Poisson model had a size of 4.51 km, which is larger than the most likely clusters detected by the STP and Bernoulli models, which have sizes of 1.59 and 4.44 km, respectively.

**Table 1:** Spatio-temporal analysis of lumpy skin disease outbreaks in dairy farms, Khon Kean province, Thailand by space-time permutation model.

**Cluster type**	**Cluster time**	**Centroid (X, Y)/radius (km)**	**O**	**E**	**O/E ratio**	**Test statistic**	***P*** **-value**
Most likely cluster	2021-06-19 to 2021-06-25	(16.593236N, 102.788555E)/1.59 km	229	8.69	4.7	180.4237	<0.001
Secondary cluster 2	2021-07-03 to 2021-07-09	(16.546286N, 102.781654E)/ <0.1 km	61	1.36	44.8	172.9565	<0.001
Secondary cluster 3	2021-05-22 to 2021-05-28	(16.343457N, 102.886328E)/3.62 km	87	6.43	13.53	147.2578	<0.001
Secondary cluster 4	2021-05-15 to 2021-05-21	(16.55907N, 102.788136E)/ <0.1 km	59	3.13	18.85	117.9548	<0.001
Secondary cluster 5	2021-06-26 to 2021-07-02	(16.61708N, 102.791554E)/ <0.1 km	79	7.63	10.35	114.2132	<0.001
Secondary cluster 6	2021-05-29 to 2021-06-04	(16.532261N, 102.823947E)/4.76 km	289	108.68	2.66	108.6684	<0.001
Secondary cluster 7	2021-05-01 to 2021-05-07	(16.506045N, 102.962927E)/ <0.1 km	27	0.27	101.22	98.06552	<0.001
Secondary cluster 8	2021-06-12 to 2021-06-18	(16.60954N, 102.795567E)/0.38 km	110	20.81	5.29	95.45388	<0.001
Secondary cluster 9	2021-05-15 to 2021-05-21	(16.583587N, 102.805018E)/ <0.1 km	45	2.39	18.85	89.86069	<0.001
Secondary cluster 10	2021-06-26 to 2021-07-02	(16.577862N, 102.789175E)/ <0.1 km	46	4.44	10.35	66.27303	<0.001
Secondary cluster 11	2021-06-26 to 2021-07-02	(16.555335N, 102.784611E)/ <0.1 km	45	4.35	10.35	64.8255	<0.001
Secondary cluster 12	2021-05-29 to 2021-06-04	(16.577353N, 102.789675E)/ <0.1 km	78	15.58	5.01	63.92709	<0.001
Secondary cluster 13	2021-06-05 to 2021-06-11	(16.623443N, 102.786429E)/0.66 km	103	31.24	3.3	52.08693	<0.001
Secondary cluster 14	2021-06-05 to 2021-06-11	(16.614593N, 102.794055E)/0.2 km	75	18.74	4	48.32974	<0.001
Secondary cluster 15	2021-06-12 to 2021-06-18	(16.560732N, 102.781424E)/0.66 km	97	32.54	2.98	42.26863	<0.001

O, observed case; E, expected case; O/E ratio, ratio of observed cases/expected cases.

**Table 2:** Spatio-temporal analysis of lumpy skin disease outbreaks in dairy farms, Khon Kean province, Thailand by Poisson model.

**Cluster type**	**Cluster time**	**Centroid (X, Y)/radius (km)**	**O**	**E**	**O/E ratio**	**RR**	**LLR**	***P*** **-value**
Most likely cluster	2021-06-05 to 2021-06-11	(16.589332N, 102.792684E)/4.51 km	635	131.95	4.81	5.97	546.8062	<0.001
Secondary cluster 2	2021-05-29 to 2021-06-04	(16.22796N, 102.759328E)/34.25 km	104	11.65	8.93	9.24	136.9307	<0.001
Secondary cluster 3	2021-05-29 to 2021-06-04	(16.547135N, 102.784079E)/ <0.1 km	68	10.92	6.22	6.36	67.86566	<0.001
Secondary cluster 4	2021-06-26 to 2021-07-02	(16.547471N, 102.779126E)/ <0.1 km	39	5.71	6.83	6.92	41.85666	<0.001
Secondary cluster 5	2021-06-19 to 2021-06-25	(16.543432N, 102.782244E)/ <0.1 km	15	2.36	6.35	6.38	15.11925	<0.001
Secondary cluster 6	2021-06-12 to 2021-06-18	(16.546014N, 102.782546E)/ <0.1 km	21	5.91	3.56	3.58	11.58984	<0.001

O, observed case; E, expected case; O/E ratio, ratio of observed cases/expected cases; RR, relative risk; LLR, Log-likelihood ratio.

**Table 3:** Spatio-temporal analysis of lumpy skin disease outbreaks in dairy farms, Khon Kean province, Thailand by Bernoulli model.

**Cluster type**	**Cluster time**	**Centroid (X, Y)/radius (km)**	**O**	**E**	**O/E ratio**	**LLR**	***P-*** **valu*e***
Most likely cluster	2021/6/5 to 2021/6/11	(16.583796N, 102.816608E)/4.44 km	525	396.47	1.32	161.35	<0.001

O, observed case; E, expected case; O/E ratio, ratio of observed cases/expected cases; LLR, Log-likelihood ratio.

**Figure 3:**
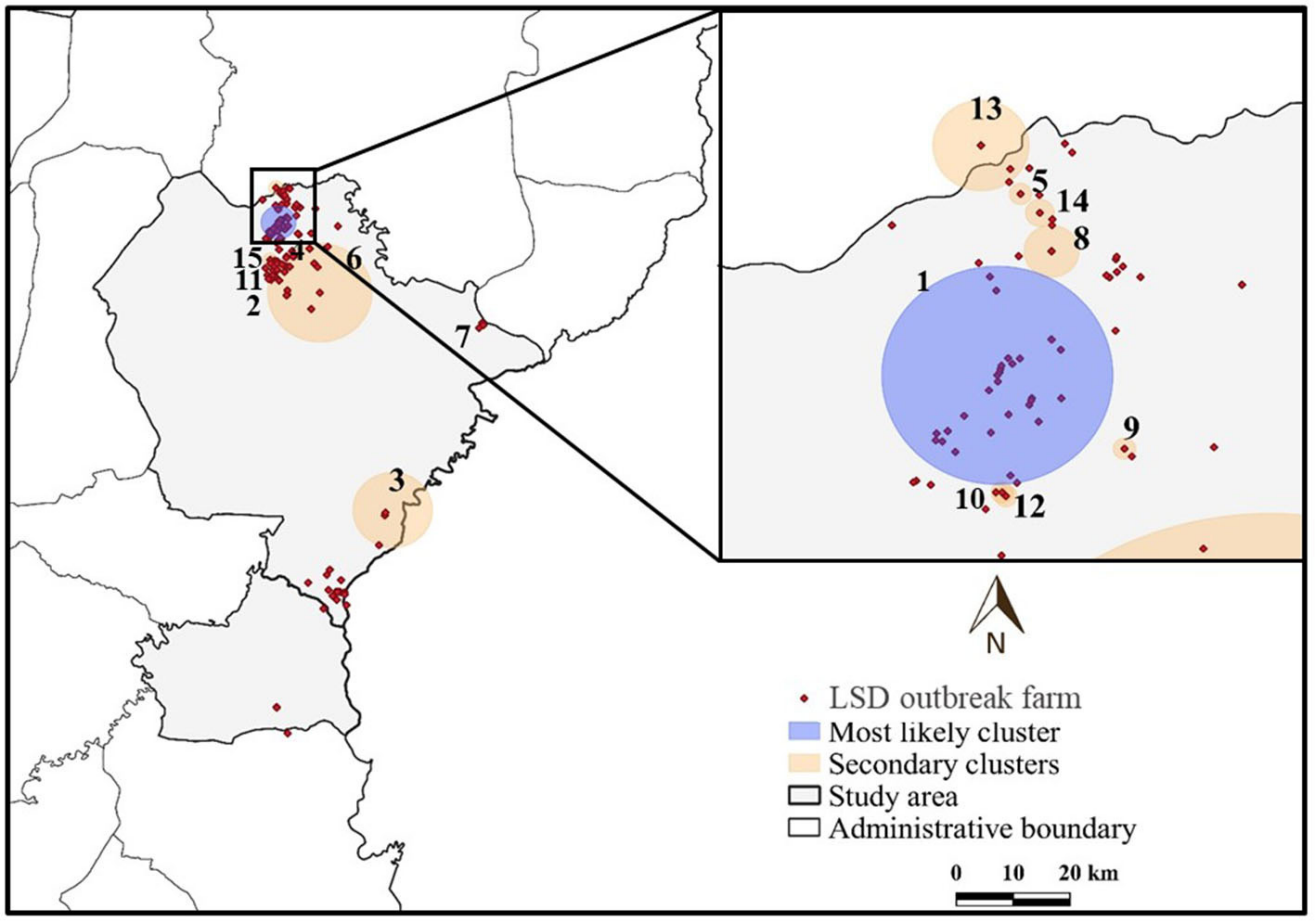
Spatio-temporal clusters of lumpy skin disease (LSD) outbreaks in dairy farms, Khon Kean province, Thailand detected by the space-time permutation model.

**Figure 4:**
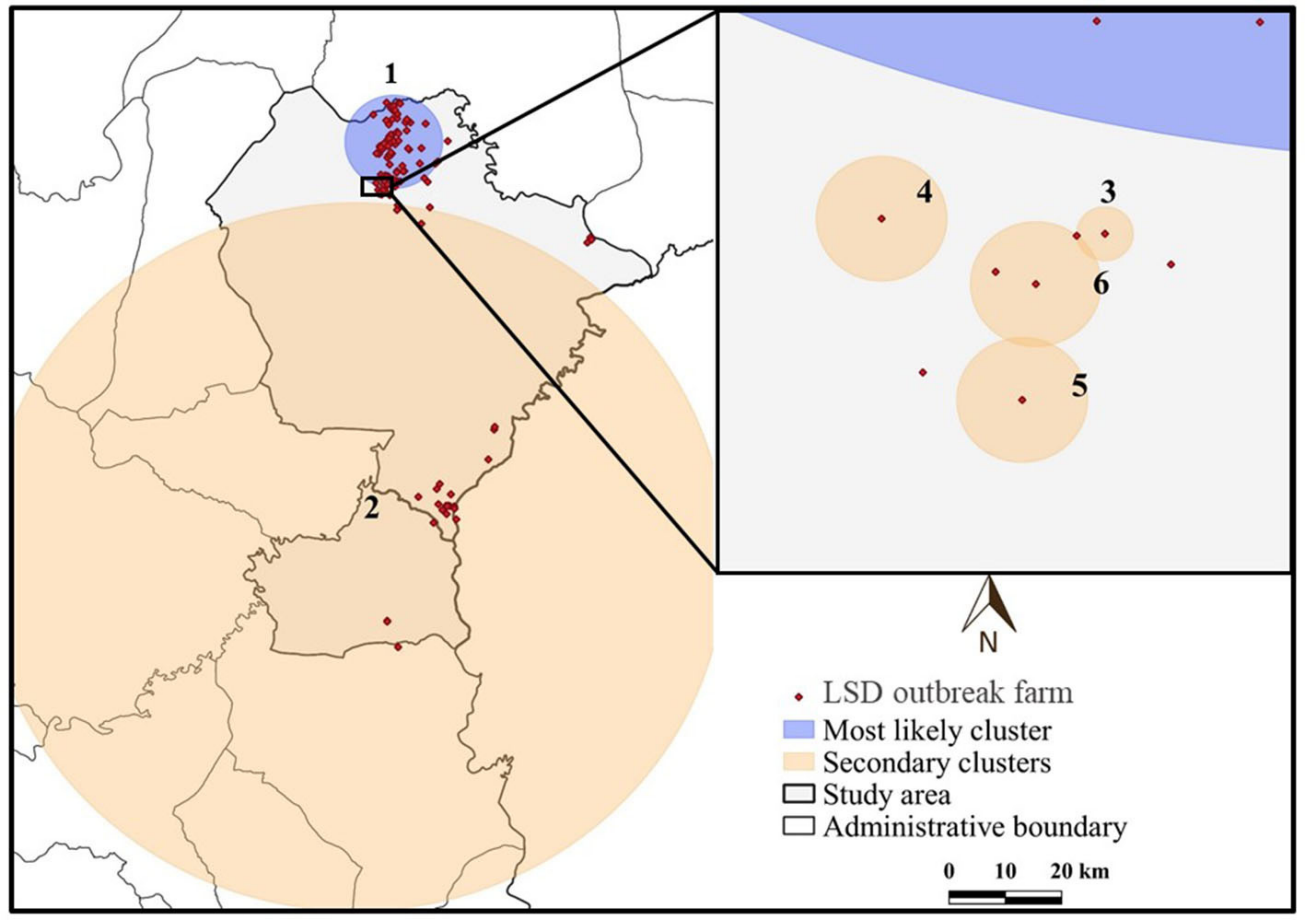
Spatio-temporal clusters of lumpy skin disease (LSD) outbreaks in dairy farms, Khon Kean province, Thailand detected by the Poisson model.

**Figure 5:**
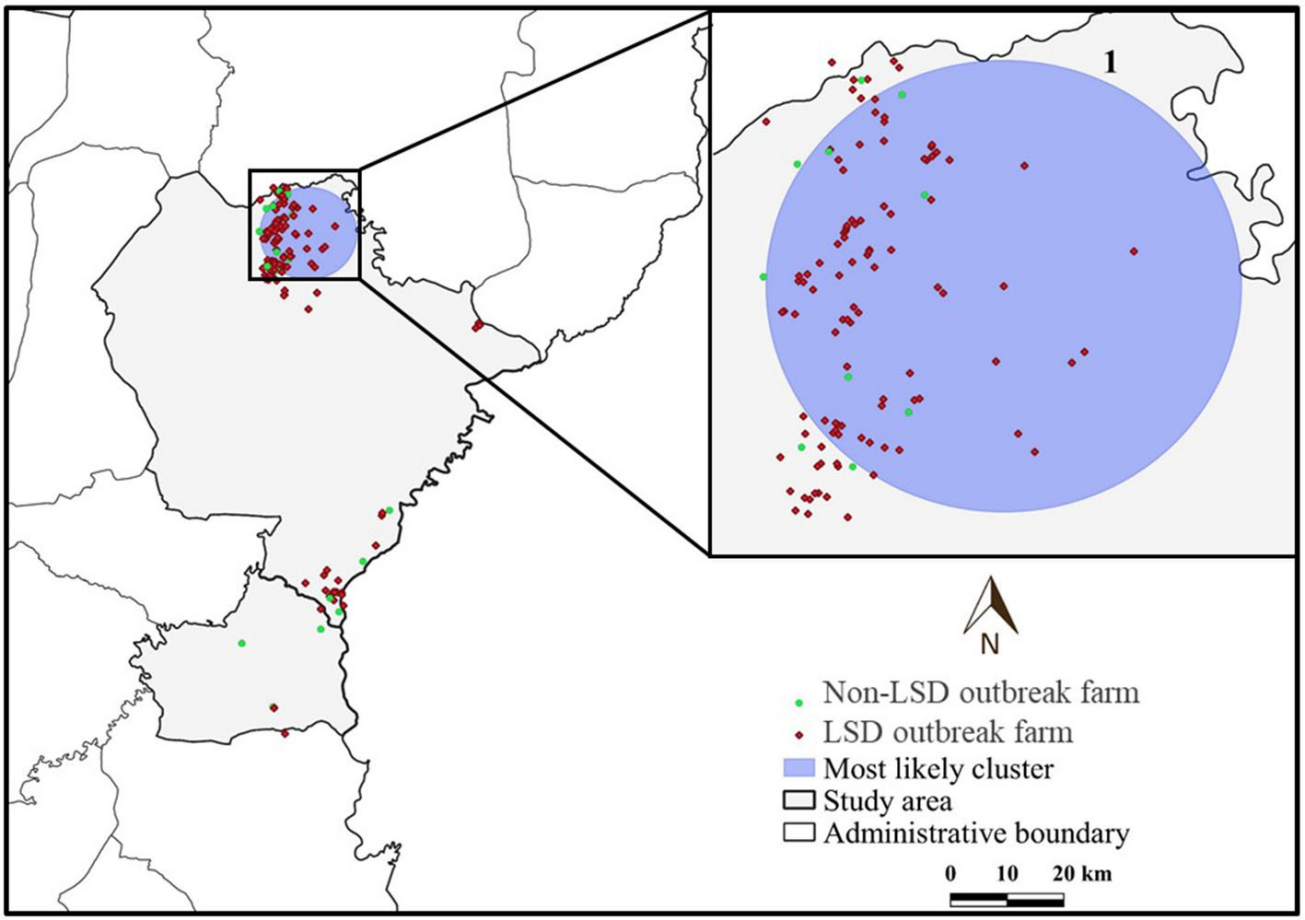
Spatio-temporal clusters of lumpy skin disease (LSD) outbreaks in dairy farms, Khon Kean province, Thailand detected by the Bernoulli model.

The most likely cluster identified by the STP model consisted of 229 LSD cases from 29 farms over a period of 7 days (Figure 3), whereas the Poisson model identified a larger cluster area consisting of 635 LSD cases from 103 farms (Figure 4) over the course of a week. The most likely clusters provided by the Bernoulli included 525 LSD cases from 79 farms and 231 dairy cattle without LSD clinical signs from 5 farms (Figure 5).

The most likely cluster from the STP model was located within the most likely cluster determined by the STP model. In other words, the same location was detected in both models, but the Poisson model included more LSD cases and covered a larger cluster area. In addition, the Poisson model detected the most likely spatio-temporal clusters earlier than the STP model. Nonetheless, the two most likely clusters were observed in June, when the largest number of LSD affected dairy farms was found. Furthermore, the Bernoulli model identified the most likely cluster observed during the same time period as the Poisson model.

The STP model identified 14 secondary clusters, 12 of which had a radius of <1 km (Table 1), whereas the Poisson model offered the largest secondary cluster (radius = 34.25 km) and 3 small clusters with a radius of <1 km (Table 2). The largest secondary cluster (Secondary cluster 6 in Table 1) derived from the STP model included a greater number of cases than those derived from the Poisson model.

## Discussion

To the best of our knowledge, this is the first study that utilized scan statistics including STP, Poisson and Bernoulli models to identify spatio-temporal clusters of LSD outbreaks in dairy farms in northeastern Thailand.

The most likely spatio-temporal clusters from all models were found in the northern part of the study area, where numerous dairy farms are aggregated. The most likely LSD clusters identified by STP were within the most likely cluster detected by the Poisson model, implying that both models suggested the same area for high LSD cases even though the temporal periods defined by both models were slightly different. Accordingly, the finding of the high number of cases and LSD-affected farms within a week suggests that LSD spreads rapidly among cattle both within and between farms.

The LSD virus can spread between cattle through close contact, insect-vectors, and other routes of transmission (3–6). In the study areas, because cattle movements among dairy farms are very low, the spread of LSD among dairy farms was less likely due to close contact between cattle from different farms. On the other hand, the spread of LSD is likely caused by insect vectors, which are abundant in most dairy farms in Thailand (37). Indeed, the finding that LSD outbreaks were found in a large number of farms within a short period of time and that several farms are aggregated in the area raises the possibility that the LSD viruses are most likely transmitted by insect vectors. This scenario was comparable to the findings of an Albanian study, which showed that if the distances between herds are short (<5 km), vector-borne contribute to the majority of LSD transmission (38). Our results are also consistent with those of a recent study conducted in Kazakhstan, which indicates that, in the absence of active animal movement, insect vectors may play a significant role in the spread of the LSD virus among cattle herds (39).

Regarding DLD control measures, DLD authorities recommended that if a farm encounters an LSD outbreak, neighboring farms within a 50 km radius should impose cattle movement restrictions, and the owners of cattle farms neighboring the outbreak farm should also take action to control such vectors (22). However, there was no guidance on the recommended size of the circle in which neighboring farms should be included. Regarding the approaches demonstrated herein, we suggested decision-makers or authorities utilize spatio-temporal models to determine the appropriate radius for the circle for each outbreak and use the results from the analyses to guide farmers. For example, for the study area, farmers who own farms within a 1-km radius of the outbreak farm should be guided to stricter insect vector control measures to prevent disease spread.

Although STP and Poisson models have been used in previous studies (23, 40), the use of the Bernoulli model for analyzing LSD outbreak data is very limited. In fact, the application of Bernoulli model to LSD outbreak data provides some advantages (41, 42). This model uses the number of cases and total population at risk from both control and LSD outbreak farms to determine clusters that include both control and outbreak farms, rather than just outbreak farms, as STP and Poisson do (41). Thus, the results from this model offer an opportunity to investigate the similarities and differences between control and LSD outbreak farms located in the same cluster (42). Due to the high herd attack rate, there are few farms without an LSD outbreak in this study. As a consequence of this, the Bernoulli model identified the most likely cluster that consisted of a limited number of non-LSD outbreak (control) farms, limiting the ability to compare potential factors associated with LSD outbreaks between non-LSD outbreak and LSD outbreak farms. However, in follow-up studies for other outbreaks where sample size is sufficient, we recommend further investigation of LSD outbreak farms (case) and non-LSD outbreak farms (control) located in the same spatio-temporal cluster to identify environmental factors, insect transmission-related factors (e.g., insect abundance and seasonality), and management practices that may be associated with outbreaks among cattle within or between farms.

It was demonstrated that the mortality and morbidity rates observed in the present study were in a range of previous reports from other countries (7, 13). These epidemiological parameters, on the other hand, were lower than those from a study conducted in beef farms in Thailand (23). This may be due to the fact that dairy farms are more intensive than beef farms, and therefore their disease prevention and control management practices are likely to be more rigorous (22). However, the differences in spatial characteristics between beef and dairy farms may also contribute to this comparison as well. For a better understanding of whether LSD affects beef and dairy cattle differently, follow up studies on this issue are necessary.

There were some limitations to the study that should be taken into consideration when interpreting the results. Even though many cattle showed LSD-like clinical signs, DLD livestock authorities collected blood and tissue samples from some cattle in some herds for further confirmation by laboratory results. Thus, the vast majority of LSD cattle cases were diagnosed based on clinical signs. However, this limitation has been encountered in many previous studies (11, 43–45). Moreover, despite the possibility that inaccurate diagnoses (such as misdiagnosis of other illnesses as LSD) in the current study may be a source of bias, this concern may not be a major issue given that the diagnoses were made by veterinarians under the supervision of the DLD livestock authorities. Furthermore, because dairy farming areas in Thailand differ geographically, the findings of this study should not be generalized to all dairy farms in the country. Indeed, epidemiological research on LSD outbreaks in other dairy farming areas with varying geographic and environmental conditions should be conducted in follow-up studies.

In this study, we demonstrated how commonly collected outbreak data can be utilized to gain additional insight into disease outbreak patterns using spatio-temporal analytical tools. We recommend incorporating the spatio-temporal analysis demonstrated herein into the current LSD surveillance system (22) to gain a better understanding of the epidemiology of LSD and generate results about hot zones or areas where resource allocations could be prioritized.

## Conclusion

This study is the first to determine the burden of LSD outbreaks in dairy farms and provide insight into the spatio-temporal analysis of disease outbreaks in the northeastern region of Thailand. The findings from this study will benefit livestock authorities in better understanding LSD epidemiology, which is important information to enhance efforts and formulate an effective control strategy for preventing future LSD outbreaks.

## Data availability statement

The raw data supporting the conclusions of this article will be made available by the authors, without undue reservation.

## Author contributions

VP, CJ, SP, and SS designed the study. VP and CJ provided oversight for the project. VP, CJ, SP, SS, and OA gathered and organized the data from different sources. VP, CJ, SS, CS, and OA analyzed the data and interpreted the results. VP, CJ, SS, and CS prepared the initial draft of the manuscript. All authors participated in the editing of the submitted article and have given their final approval.

## Funding

This research was funded by National Research Council of Thailand (NRCT), Thailand.

## Conflict of interest

Author SP was employed by company Khon Kaen Dairy Cooperative. The remaining authors declare that the research was conducted in the absence of any commercial or financial relationships that could be construed as a potential conflict of interest.

## Publisher's note

All claims expressed in this article are solely those of the authors and do not necessarily represent those of their affiliated organizations, or those of the publisher, the editors and the reviewers. Any product that may be evaluated in this article, or claim that may be made by its manufacturer, is not guaranteed or endorsed by the publisher.
